# Reviewed article: Silva AGV, Miguez FGG, Siqueira LLC, Leite FMC. Perception of stress and its association with socioeconomic, behavioral and clinical characteristics of women: a cross-sectional study in Vitória, Espírito Santo. Epidemiol Serv Saude. 2025:34;e 20240224

**DOI:** 10.1590/S2237-96222025v34e20240224.a

**Published:** 2025-08-04

**Authors:** Maryana Carmello da Costa

**Affiliations:** FOUSP

**Table:** 

Rating	Excellent	Good	Average	Below average	Poor
Interest		✓			
Quality			✓		
Originality		✓			
Overall			✓		

**Table:** 

Questionnaire	Yes	No	Not applicable
Does the manuscript contain new and significant information to justify publication?	✓		
Does the Abstract (Summary) clearly and accurately describe the content of the article?	✓		
Is the problem significant and concisely stated?	✓		
Are the methods described comprehensively?	✓		
Are the interpretations and conclusions justified by the results?	✓		
Is adequate reference made to other work in the field?	✓		

## Manuscript Structure

Length of article is: Too long

Number of tables is: Adequate

Number of figures is: Adequate

Please state any conflict(s) of interest that you have in relation to the review of this paper (state “none” if this is not applicable): None

## Comments

Em relação ao título do manuscrito, indique o desenho do estudo no título ou no resumo, com termocomumente utilizado.

Em relação ao delineamento escolhido, justifique a escolha do delineamento do estudo e explique como eleajudará a responder à pergunta de pesquisa.

Nas tabelas, conforme orientação ao autor, apresente dados que se complementam em coluna única, comofrequência absoluta e relativa: “| N (%) |” e medida de associação e de dispersão: “| RP (IC95%) |”. Evitecolunas com recíproco de dado já apresentado (informação redundante), por exemplo: somente uma colunacom a distribuição absoluta e relativa da doença, sem necessidade de outra coluna com a distribuição entresaudáveis.

Revise o resumo e descreva detalhadamente e com clareza os métodos do estudo e como os resultadosforam obtidos, trazendo mais informações. Apresente os principais resultados da pesquisa, com númerosque apoiam as afirmações. A conclusão deve responder ao objetivo e ter mais informações.

Separe o texto dos resultados por parágrafo e organize, as ideias devem ser apresentadas de formaorganizada e lógica.

O texto na discussão precisa ser revisado para garantir precisão e objetividade na linguagem. Separe o textopor parágrafos e organize para ter uma maior fluidez no texto.

